# Adaptive Indoor Area Localization for Perpetual Crowdsourced Data Collection

**DOI:** 10.3390/s20051443

**Published:** 2020-03-06

**Authors:** Marius Laska, Jörg Blankenbach, Ralf Klamma

**Affiliations:** 1 Geodetic Institute and Chair for Computing in Civil Engineering & Geo Information Systems, RWTH Aachen University, Mies-van-der-Rohe-Str. 1, 52074 Aachen, Germany; blankenbach@gia.rwth-aachen.de; 2 Advanced Community Information Systems Group (ACIS), RWTH Aachen University, Lehrstuhl Informatik 5, Ahornstr. 55, 52074 Aachen, Germany; klamma@dbis.rwth-aachen.de

**Keywords:** indoor localization, area localization, crowdsourcing, fingerprinting, deep learning

## Abstract

The accuracy of fingerprinting-based indoor localization correlates with the quality and up-to-dateness of collected training data. Perpetual crowdsourced data collection reduces manual labeling effort and provides a fresh data base. However, the decentralized collection comes with the cost of heterogeneous data that causes performance degradation. In settings with imperfect data, area localization can provide higher positioning guarantees than exact position estimation. Existing area localization solutions employ a static segmentation into areas that is independent of the available training data. This approach is not applicable for crowdsoucred data collection, which features an unbalanced spatial training data distribution that evolves over time. A segmentation is required that utilizes the existing training data distribution and adapts once new data is accumulated. We propose an algorithm for data-aware floor plan segmentation and a selection metric that balances expressiveness (information gain) and performance (correctly classified examples) of area classifiers. We utilize supervised machine learning, in particular, deep learning, to train the area classifiers. We demonstrate how to regularly provide an area localization model that adapts its prediction space to the accumulating training data. The resulting models are shown to provide higher reliability compared to models that pinpoint the exact position.

## 1. Introduction

In recent years, the usage of location-based services (LBS) has experienced substantial growth. This is mostly caused by the wide adoption of smartphones with the ability to reliably track a user’s location. Global Navigation Satellite Systems (GNSS), such as the Global Positioning System (GPS), are the dominant technology to enable LBS, since they offer accurate and reliable localization performance. However, GNSS do not provide sufficient availability and reliability inside buildings, since the satellite signals are attenuated and scattered by building features. This drawback has led to the development of various alternative indoor localization systems [1], which utilize a spectrum of techniques and technologies. Until today, there is not any gold standard for indoor localization, which can be stated as the main issue that has prevented indoor LBS from developing their full potential [2].

Indoor localization systems can serve different purposes. In monitor-based systems, the location of a user or entity is passively obtained relative to some anchor node [1]. This can be utilized, for example, to enhance the energy efficiency of buildings by automatically switching-off lighting and heating/cooling in empty rooms [3,4]. In contrast, in device-based systems, the location information is obtained from a user-centric perspective [1], which can be utilized, for example, to enable navigation [5,6]. A variety of technologies and approaches are present in the field of indoor localization. Comprehensive overviews are given in [1,7,8,9,10]. In general, indoor localization systems can be grouped into (1) autonomous, (2) infrastructure-based and (3) hybrid systems. Autonomous systems apply inertial navigation [7]. In infrastructure-based systems, it can be differentiated between (2.1) analysis of signal propagation to dedicated transmitting stations and (2.2) scene analysis (fingerprinting) [11]. The former utilizes proximity, lateration or angulation measurements to estimate the user’s location. This requires line-of-sight and knowledge about the location of the stations. In contrast, fingerprinting does not rely on either. Instead, in an offline phase, the scene is scanned at certain reference points with a sensing device (e.g., smartphone). The observed sensor values at each reference point form so-called fingerprints. Using supervised machine learning (ML), a mapping from fingerprints to locations is learned, which is utilized to estimate the location for unseen fingerprints during online localization. Fingerprinting leverages existing infrastructure, which reduces upfront deployment cost. However, the accuracy of the system strongly depends on the quality of the offline site survey and the up-to-dateness of the fingerprint database.

A crowdsourced site survey has been proposed to partition the collection among several participants and thus reduces the manual labeling effort [12,13,14]. Users either explicitly tag a fingerprint with a location, or the label is implicitly inferred by the system. The decentralized collection comes with the cost of heterogeneous data, which include among others device heterogeneity, labeling noise and an unequal spatial training data distribution [15].

Area localization can be applied in settings with imperfect data to achieve reliable positioning guarantees [16]. The problem is simplified such that the goal becomes to predict the right area instead of pinpointing the exact location. Existing area localization solutions employ a static segmentation into areas that is independent of the available training data [17,18,19,20]. This approach is not applicable for crowdsoucred data collection, since it features an unbalanced spatial training data distribution that changes over time. A segmentation is required that utilizes the existing training data distribution and adapts when new data is accumulated. The amount and shape of the areas, in particular, the richness of training data per area, affect the accuracy of classification models, which we subsequently call model *performance*. In addition, the expressive power is determined by the segmentation. If a model predicts one of few but large classes, the information gain of the user is lower compared to models that predict one of many smaller areas. We call the expressive power of the model that is determined by the segmentation *expressiveness*. Since crowdsourced data is expected to be generated continuously, the segmentation into areas as well as the successive classification model can be continuously improved. The challenge is, therefore, to continuously find a model with the right balance between expressiveness and performance given the most recent crowdsourced map coverage.

The main contributions of this paper are summarized as follows:

- We introduce the concept of adaptive area localization to enable area classification for crowdsourced data that are continuously generated.
- We propose the idea of data-aware floor plan segmentation to compute segmentations that benefit subsequent classification. We present a clustering-based algorithm that determines such a segmentation with adjustable granularity.
- We formulate a metric to compare various area classifiers, such that the model, providing the optimal balance between expressiveness and performance, can be selected. This allows for automatic model building and selection in the setting of continuous crowdsourced data collection.
- We provide a comprehensive experimental study to validate the concepts on a self-generated and a publicly available crowdsourced data set.

The rest of the paper is organized as follows: we introduce related work in Section 2 focusing on crowdsourced data collection, area classification and deep learning. Subsequently, Section 3 introduces the proposed concepts of adaptive area classification in detail. In Section 4 we present the *locally dense cluster expansion (LDCE)* algorithm for computing floor plan segmentations with adjustable granularities that are based on the available training data. Section 5 covers details regarding machine learning model building for area classification. In Section 6 the proposed concepts are evaluated on a self-generated as well as a publicly available crowdsourced data set. Finally, we discuss our findings in Section 7 and draw conclusion in Section 8.

## 2. Related Work

Fingerprinting-based indoor localization commonly utilizes a two stage approach. In the offline phase, radio frequency (RF) signals are collected at certain reference points and tagged with the position of collection. An algorithm is used to find a mapping from unknown fingerprints to locations. This algorithm is then applied during the online phase to localize an RF device [21]. The RF technology of choice for fingerprinting is commonly WLAN, however, solutions have been proposed that utilize alternative RF technologies such as LTE [22]. The most common approach for constructing a WLAN fingerprint is the received signal strength (RSS), which can be used either directly [23,24] or after feature extraction [25,26,27,28]. Recent studies on fingerprinting also incorporate channel state information (CSI) as input data in order to obtain more accurate prediction results [29,30,31]. The underlying assumption states that RSS values do not exploit the subcarriers in an orthogonal frequency-division multiplexing (OFDM). Therefore, CSI contains richer multipath information [32], which is beneficial for training complex models. However, obtaining CSI data is only achievable with certain Wi-Fi network interface cards (NIC) and thus is currently not suitable for smartphone based data collection like crowdsourcing. In this work, we focus on classical WLAN fingerprinting and utilize the RSS of scanned access points to construct the radio frequency map.

### 2.1. Crowdsourcing

Several approaches have been proposed to reduce the manual labeling effort during crowdsourced data collection for Wi-Fi fingerprinting [12,13,14]. Rai et al. [12] were among the first to present a probabilistic model to infer the position of implicitly collected fingerprints. They periodically collected the RSS together with the timestamps of collection. Simultaneously, the system tracks the user utilizing a particle filter. After convergence, the path information is used to annotate the RSS measurements with a location. Radu and Marina [13] additionally integrated activity recognition and Wi-Fi fingerprinting via a particle filter to detect certain anchor points, such as elevator or stairs. He and Chan [33] utilized proximity information to Internet-of-things (IoT) sensing devices and the initially sparse RSS radio map to label fingerprints during implicit crowdsourcing. The IoT devices can be fixed, such as installed beacon transmitters or moving (smartphones of other participants). Santos et al. [14] utilized pedestrian dead reckoning (PDR) techniques to reconstruct the movements of users and classified the resulting trajectories using Wi-Fi measurements. Similar segments have been identified using an adaptive approach based on geomagnetic field distance. Finally, floor plans were reconstructed through a data fusion process and the collected Wi-Fi fingerprints were aligned to physical locations. Zhou et al. [34] abstracted the indoor maps as semantics graph. Crowdsourcing trajectories were mapped to the floor plan by applying activity detection and PDR. The annotated trajectories have been utilized to construct the radio map. Based on unfixed data collection, Jiang et al. [35] proposed the construction of a probabilistic radio map, where each cell was assigned a probability density function (PDF) instead of a mean value as in classical site survey approaches. Wei et al. [18] utilized the knowledge of location during the payment process inside the shops of a mall. They utilized this to annotate collected fingerprints with the current shop to build a hierarchical classification model that provides shop-level localization. In contrast to probabilistic fingerprint annotation, unsupervised learning can be utilized to obtain labeled Wi-Fi fingerprints [36,37]. Jung and Han [37] utilized unsupervised learning to infer the location of access points together with a path loss model and optimization algorithm, which they presented in [36]. They investigated how to adaptively recalibrate the resulting map to avoid performance degradation of downstream localization models.

Besides the reduction of labeling effort when collecting data via crowdsourcing, there are several additional challenges that have to be considered. Ye and Wang [15] identifed four major problems, which are:

- Inaccurate position tags for crowdsourced fingerprints that might occur during manual labeling of non-experts or are caused by automatic labeling via probabilistic models.
- The fluctuating dimensionality of RSS signals caused by varying numbers of hearable access points for various locations.
- The device heterogeneity that causes RSS to differ across various devices for the same measurement position.
- The nonuniform spatial data distribution, meaning that some areas feature a larger amount of data, while for others no data was collected.

They constructed device-specific grid fingerprints utilizing clustering-based algorithms. For sparse areas fingerprints are interpolated and finally, the samples from several devices are fused to obtain device independent grid fingerprints. Yang et al. [25] additionally identified the short measurement time of crowdsourced sample collection as a typical problem. They utilized the fact that the most-recorded RSS does not differ much, irrespective of the length of measuring, to extract a characteristic fingerprint. In a follow up work, Kim et al. [26] evaluated the system in a case study and demonstrated its effectiveness. Pipelidis et al. [38] proposed an architecture for cross-device radio map construction via crowdsourcing. They utilized data labeled via a simultaneous localization and mapping (SLAM)-like algorithm. The RSS values between devices were calibrated via reference measurements at several landmarks. The data was clustered and subsequently used for classification of areas.

### 2.2. Area Localization

In contrast to localization systems that aim at pinpointing the exact position of a user, the concept of area classification only focuses on estimating the current area of the user, such as the office room or the shop inside a mall. This is particularly suitable for large scale deployments or in situations where the data quality does not allow for accurate localization.

Lopez Pastor et al. [17] evaluated a Wi-Fi fingerprinting-based indoor localization system inside a medium sized shopping mall. The system is meant for providing shop-level accuracy, while minimizing the deployment cost and effort. Data is collected by randomly walking in predefined areas, such that all data can be labeled with the corresponding shop. The authors claim that the achieved system performance is sufficiently independent of the device and does not deteriorate over time. Wei et al. [18] adopted a similar approach. They utilized the fact that during payment inside a shop, the location of the user is known. This can be used to annotate Wi-Fi fingerprints collected while paying. The obtained fingerprints can be utilized for shop-level position estimation. Rezgui et al. [19] proposed a variation of a support vector machine (SVM) (normalized rank based SVM) to address the problem of hardware variance and signal fluctuation of Wi-Fi based localization systems. The system achieves room level prediction accuracies. He et al. [16] compared the performance of various classification models, such as SVM, artificial neural network (ANN) and deep belief network (DBN) for various test sites. They addressed the identification of floors, indoor/outdoor and buildings. In a recent follow up work [39], they also tackled the inside/outside region decision problem and propose solutions for missing AP detection and fingerprint preprocessing. Liu et al. [20] proposed an algorithm for probability estimation over possible areas. By adopting the user’s trajectory and existing map information, they eliminate unreasonable results. The partitioning of the map into areas is done manually based on the different rooms and offices.

### 2.3. Deep Learning for Fingerprinting

Fingerprinting-based indoor localization can be formulated as standard supervised learning problem. It can be modeled as regression problem with the goal to predict the exact position, or as a classification task on predetermined areas. Due to the recent success of deep learning in areas such as image processing or speech recognition, the application of deep models for fingerprinting-based indoor localization has gained attention recently. Nowicki and Wietrzykowski [40] applied stacked autoencoders combined with a feed forward neural network for building and floor prediction. Xiao et al. [23] compared SVM and a deep neural network (DNN) on various publicly available data sets and propose a data augmentation schema as well as an approach for transfer learning. Adege et al. [28] applied regression analysis to fill missing RSS values and utilize linear discriminant analysis for dimensionality reduction. Finally, feed forward neural networks are applied to tackle the regression and classification problem. Kim et al. [41] formulated the problem as multi-label classification problem to predict the building, floor and position with a single network with minimal performance degradation. Mai et al. [42] utilized a convolutional neural network (CNN) on raw RSS data by applying the convolution on time-series data. The data is artificially constructed by combining measurements within a certain cell size that have been captured in temporal intervals not exceeding a certain threshold. By constructing an image of the RSS vector, CNNs that are predominantly used for image classification can be applied. Mittal et al. [27] filtered access point signals that have a low Pearson Correlation Coefficient (PCC) between the access point values and the location vector. The remaining RSS vector is transformed into an image matrix by multiplying each access point vector with the obtained correlation values and arranging as matrix with zero padding. Sinha et al. [24] simply arranged the RSS vector as a matrix to train a standard CNN image classifier. They proposed a data augmentation scheme where single values of the RSS vector are replaced by random values sampled from the interval of the difference of the actual value and the access point mean value.

## 3. Adaptive Area Classification for Crowdsourced Data

In the following section, we introduce our approach to adaptive area classification. We describe the concept overview and introduce relevant notations. Subsequently, a floor plan segmentation is formally defined and classification models for indoor localization are described. Finally, we propose a novel metric called ACS, which is utilized to select area classifiers with respect to the optimal balance between expressiveness and performance.

### 3.1. Concept Overview

The performance of Wi-Fi fingerprinting-based indoor localization systems heavily relies on thorough and up-to-date site survey data. Crowdsourced training data collection continuously provides fresh data, but suffers from poor data quality. Several approaches suggest to maintain an up-to-date radio map, which stores a representative fingerprint or a probabilistic distribution for predefined locations, regions, or grid cells [33,35,43]. Missing data for certain locations prohibits equal radio map quality at all areas. This is solved by either enlarging the areas of the radio map or by interpolating fingerprints for sparsely covered areas [15]. The update of such a radio map is a complicated process, since its granularity is static. However, the spatial distribution of available training data is expected to shift over time. Therefore, instead of maintaining a radio map with characteristic fingerprints for predefined areas, we store the entire training data with the noisy position tags. At regular intervals, we dynamically subdivide the floor plan into areas based on the richness of available training data. The training data, which are originally annotated with noisy position tags, are labeled with the corresponding areas based on the computed floor plan segmentation. This enables training of standard supervised machine learning classifiers that predict the correct area. In order to quantify the gain of such an area classifier, two metrics can be utilized.

- The *expressiveness* measures the information gain of the user, which is mainly influenced by the extent of each individual area and the total coverage of the model.
- The *performance* indicates how reliably the model predicts a certain area.

**Figure 1:**
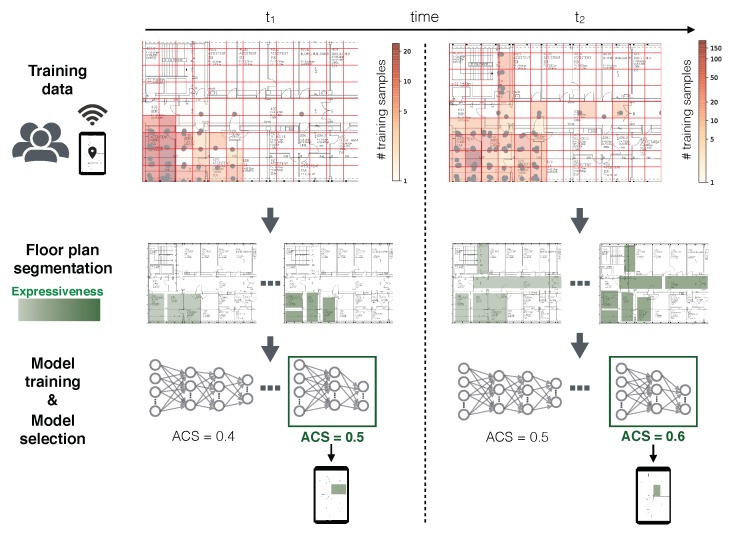
Concept of adaptive area classification for crowdsourced map coverage.

The two metrics are inversely proportional. That means a fine segmentation (high expressiveness) negatively affects the performance of the model and vice versa. We assume that fresh crowdsourced training data is accumulated over time. This enables updates of the floor plan segmentation and the successive area classifier. The workflow for continuously providing area localization models, where the prediction space adapts to the new training data, is illustrated in Figure 1. Over time, the map gets covered with an increasing amount of training data, which is illustrated in the top row of Figure 1. At regular intervals, the goal is to provide an optimal indoor area classification model based on the current map coverage. This process includes the automatic floor plan segmentation into areas and the training of an ML model. Several floor plan segmentations can be determined that influence the expressiveness of the ML model and for each of these segmentations several ML models can be learned. For each epoch, the best combination of segmentation and model is selected. This is done with respect to a metric, called area classification score (ACS). The ACS balances expressiveness and performance and is introduced in Section 3.5.

### 3.2. Data Notations

In the following, we introduce the formal notations that are subsequently used. We assume that at a certain point in time, a set of *N* labeled training data tuples (fingerprints) $FP=\{fp_{n}=\left(x_{n},p_{n},t_{n}\right)\}$ for n=1,…,N has been collected for a given indoor map. Each fingerprint fp consists of a *M*-dimensional feature vector $x={\left(x_{1},\ldots ,x_{M}\right)}^{T}$ and is tagged with a position $p_{n}={\left(p_{x},p_{y}\right)}^{T}$ in two dimensions and the corresponding timestamp $t_{n}$ of collection. In the following we focus on Wi-Fi fingerprinting, such that each entry of the vector is the RSS value of the corresponding access point and *M* is equal to the total amount of access points that are observable for the map. Since not all access points are hearable at all locations, x contains missing entries, which have to be considered during further processing of the data.

### 3.3. Floor Plan Segmentation for Area Classification

In order to train a classification model, we have to find a floor plan segmentation that assigns each fingerprint tuple (x,p,t) to one of the K areas or classes, $C_{k}$ for k=1,…,K. A floor plan segmentation determines a mapping $SEG:C_{k}\to A_{k}$, where $A_{k}$ might be any two-dimensional shape, such as a rectangle. Given such a mapping SEG, we can label each fingerprint with the class label of the area it is located in. For a given segmentation SEG, we obtain the transformed set $FP_{SEG}=\left\{\left(x_{n},c_{n}\right)\right\}$, where $c_{n}\in \left\{1,\ldots ,K\right\}$ and $c_{n}=k\Leftrightarrow p_{n}\;\mathrm{lies}\;\mathrm{within}\;A_{k}$. The goal is now to find a classifier $C:x\to c_{k}$ that determines the correct area of the floor plan segmentation for an unknown RSS fingerprint. We have now arrived at the standard formulation of a supervised learning problem, in particular, a classification problem.

### 3.4. ML Models for Area Classification

Given the transformed set of fingerprints $FP_{SEG}=\left\{\left(x_{n},c_{n}\right)\right\}$ for a segmentation SEG, we can utilize any standard ML classification model that learns to predict the unknown class $c_{k}$ for a fingerprint x. We can either construct a discriminant function that directly assigns a class to an unknown fingerprint, or we model the conditional probability distribution $p(c_{k}|x)$ [44]. SVMs depict a typical discriminant model used in the domain of indoor localization, while with DNNs, it is possible to model $p(c_{k}|x)$. Both models are utilized in the experimental study (Section 6) as classifiers for the transformed fingerprint sets $FP_{SEG}$.

### 3.5. Area Classification Score

In order to properly quantify the quality of the learned area localization model (combination of segmentation and trained classifier), we have to simultaneously investigate the model’s expressiveness as well as its performance. The expressiveness is influenced by the total extent of covered area as well as the size of each individual area. We state that the expressiveness of a model is higher if it predicts classes associated with smaller areas. However, the benefit of a narrow prediction area vanishes if the performance for that specific class, for example the accuracy, is poor. To capture this interplay, we have to look at each predicted class of the classifier individually. We define $area_{k}$ as the surface area of the area $A_{k}$ that belongs to class $C_{k}$. On an individual class level, we define the expressiveness of class $C_{k}$ as:

(1) $$exp_{\lambda }\left(C_{k}\right)=\frac{area_{min}}{area_{k}^{\lambda }}\;,$$

where $area_{min}$ is the minimal extent that an area might have by definition (set to $1m^{2}$ in the following) and λ is a parameter to adjust the slope of the function. Additionally, a performance metric is required, which measures the accuracy of the model on a class level. We choose the $F_{1}$ score, since we are equally interested in precision and recall. Let $F_{1}\left(C_{k}\right)$ be the class-based $F_{1}$ score for class $C_{k}$, evaluated on a separate test set. The chosen metrics for expressiveness and performance reside in the interval [0,1], such that we can multiply them to obtain a value in [0,1], which would be optimal, if the predicted class has the minimal extent of $1m^{2}$ and a $F_{1}$-score of 1 on the test set. In order to account for the total covered area, we take the weighted mean of the product of expressiveness and performance using the area of each class. We finally arrive at:

(2) $$ACS=\frac{1}{area_{tot}}\sum_{k=1}^{k}F_{1}{\left(C_{k}\right)}^{\mu }\cdot exp_{\lambda }\left(C_{k}\right)\cdot area_{k}\;,$$

which we call area classification score (ACS) in the following. The expressiveness term (1) regulates how much the class score adds to the weighted mean. For λ=0, the regularization term vanishes, such that the area size of the specific class has no influence on the amount that is added to the mean. This means that two localization models with constant class-wise classification performance $\bar{F_{1}}$ achieve the same score if they cover the same area $area_{cov}$, independent of the amount of classes and their individual size:

(3) $$ACS_{\lambda =0}=\frac{area_{cov}}{area_{tot}}\cdot \bar{F_{1}}\;.$$

It follows that if λ approaches 0, the metric becomes less sensitive to the individual area sizes. With respect to models covering a similar extent of the map, those that provide a higher performance will be rated higher, independent of the number and individual size of their areas. The closer λ gets to 1, the higher is the influence of individual area sizes. High performance on broad areas will not add much to the weighted mean, since they are downscaled by the expressiveness factor. As a consequence, models with finer segmentations score higher, since the influence of area regularization outweighs the performance factor. For λ=1, the score is only sensitive to the amount of total segments. The ACS becomes

(4) $$ACS_{\lambda =1}=\frac{1}{area_{tot}}\sum_{k=1}^{k}F_{1}{\left(C_{k}\right)}^{\mu }\;,$$

which will be higher for finer segmentations given that the same total extent of the map is covered. The parameter μ can be utilized for fine tuning. By setting it larger than 1, models with overall low performance are penalized. We found that λ has a greater impact on the model selection and suffices for our use-cases. Therefore, μ is set to 1 during subsequent application of the ACS.

**Figure 2:**
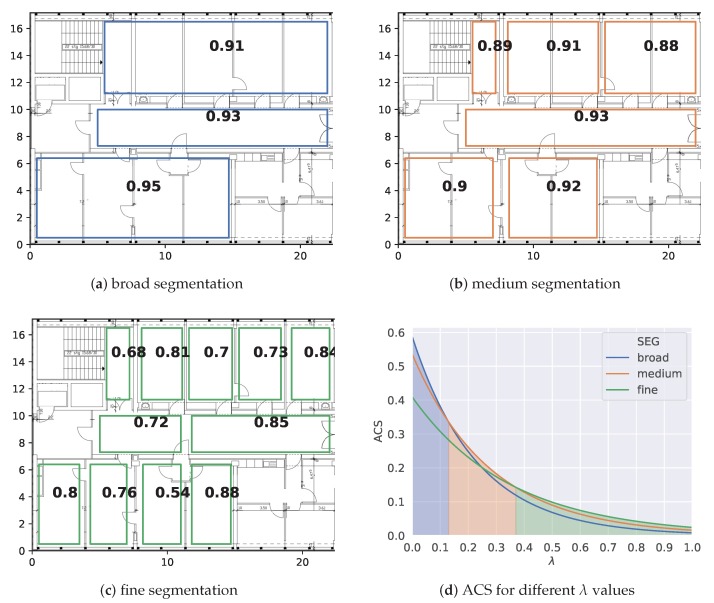
Illustration of the impact of λ on the ACS for pool of example models.

Figure 2 emphasizes how the parameter choice of λ affects the ACS for three artificial segmentations (a–c). The rectangular boxes represent the prediction areas of the classifier and the numbers show the class-wise $F_{1}$ scores on a separate test set. We stated that λ influences the expressiveness. In particular, the closer the value gets to 1, the more each individual class score is downscaled by the size of its area. As a consequence, a low λ value targets high performant models with lower expressiveness and a high λ value selects models with high expressiveness and lower performance. Given the three segmentations (a–c), we plot the ACS for all possible choices of λ in Figure 2d to investigate which model achieves the highest score (illustrated by the color below the curve). As expected, the broad segmentation (a) is selected for low lambda values (0–0.13), the medium segmentation (b) is chosen for values (0.13–0.37) and the fine segmentation (c) is chosen for higher values (0.37–1). In practice, a pool of models is trained such as (a–c). The λ parameter is fixed, such that the best scoring model is determined. If the model does not adhere to the required use case requirements, λ can be adjusted accordingly, such that a different model is obtained.

## 4. Floor Plan Segmentation Algorithms

In order to train an area classification model, we have to determine a mapping from areas to classes that we defined as floor plan segmentation. If we neglect the underlying training data distribution, we end up with segmentations where certain classes feature few to zero fingerprint samples. This results in unsatisfying classification performance. The goal should be to leverage the knowledge about available training data to compute a segmentation that benefits subsequent classification but still provides the best possible expressiveness. We call such a segmentation *data-aware floor plan segmentation* and present an algorithm for this purpose in the following.

### Locally Dense Cluster Expansion (LDCE)

In the following, we introduce the LDCE algorithm that computes a floor plan segmentation, in particular, a mapping $SEG:C_{k}\to A_{k}$ that assign each class $C_{k}$ a shape $A_{k}$. Given SEG, we can label fingerprints (x,p,t) with the class that belongs to the area $A_{k}$ in which p is located. Let $FP=\{fp_{n}=\left(x_{n},p_{n}\right)\}$ for n=1,…,N be a set of training data, we cluster the observations and determine the shapes $A_{k}$ based on the position labels of the resulting cluster members.

Initially, we detect a set of locally dense base clusters. This serves two purposes: (1) observations that are densely connected to a certain degree should not be separated and (2) fingerprints that are not part of any initially dense cluster should be considered as noise. Both conditions are fulfilled when applying a standard density based clustering algorithm such as the density-based spatial clustering of applications with noise (DBSCAN) algorithm.

The resulting base clusters are subsequently expanded. Each round the closest clusters are determined and merged. Resulting clusters that contain the required amount of stop_size members are deleted from the expansion set and added to the set of final clusters. This process is continued until either no clusters are present in the expansion set, or the smallest minimal distance exceeds the maximal allowed merging distance max_eps. Remaining clusters with fewer than stop_size members are postprocessed. By setting the minMembers parameters lower than stop_size, those clusters having at least minMember members are added to the set of final clusters. All other remaining clusters are added to the closest final cluster.

This routine yields clusters with definable bounds for the amount of members. Since clusters with more than stop_size members are excluded from the merging phase, any merged cluster might have at most 2·stop_size members. However, besides the amount of available training data per segment, we require a reasonable segmentation that adheres to the physical floor plan structure. In particular, segmentations should minimize spreads across multiple walls if possible. Furthermore, since the feature vector of subsequent classification consists of the RSS vector, the similarity in RSS signal space should be considered during the segmentation phase. The approach we propose achieves this by constructing a particular distance function between fingerprints and clusters of fingerprints that is used in the previously described algorithm. Given two fingerprints $fp_{u}=\left(x_{u},p_{u}\right)$ and $fp_{v}=\left(x_{v},p_{v}\right)$, we define their distance as:

(5) $$dist\left(fp_{u},fp_{v}\right)=\left|\right|p_{u}-p_{v}{\left|\right|}_{2}+\theta \cdot |W_{p_{u},p_{v}}\left|+\zeta \cdot |\right|x_{u}-x_{v}{\left|\right|}_{2}\;,$$

where $W_{p_{u},p_{v}}$ is the set of walls between $p_{u}$ and $p_{v}$. Note that the main distance factor is the Euclidean distance between the position labels, while the difference between RSS vectors and the number of conflicting walls are used to penalize this base distance. The distance between clusters is based on centroid distance. We add another penalty term to account for final clusters that might lie between merging clusters. Let $C_{i}$ and $C_{j}$ be two clusters, ${\bar{p}}_{i}$, ${\bar{p}}_{j}$ the average position labels and ${\bar{x}}_{i}$, ${\bar{x}}_{j}$ the average RSS vectors, the distance is then given by:

(6) $$dist\left(C_{i},C_{j}\right)=\left|\right|{\bar{p}}_{i}-{\bar{p}}_{j}{\left|\right|}_{2}+\theta \cdot |W_{{\bar{p}}_{i},{\bar{p}}_{j}}\left|+\zeta \cdot |\right|{\bar{x}}_{i}-{\bar{x}}_{j}{\left|\right|}_{2}+\eta \cdot \left|C_{i,j}\right|\;,$$

where $C_{i,j}$ is the subset of final clusters, such that $C_{f}\in C_{i,j}\Leftrightarrow \exists fp_{f}\in C_{f}|p_{f}$ within $bounds({\bar{p}}_{i},{\bar{p}}_{j})$. In order to prevent merging of far distant clusters, with respect to the penalized distance function, we set a threshold max_eps on the maximal allowed merging distance of two clusters. Note that the choice of max_eps determines the maximal amount of allowed walls between two merging clusters. If we choose max_eps=θ·x+δ, it holds that for any δ<θ, there will be at most x−1 separating walls between any merging cluster.

**Figure 3:**
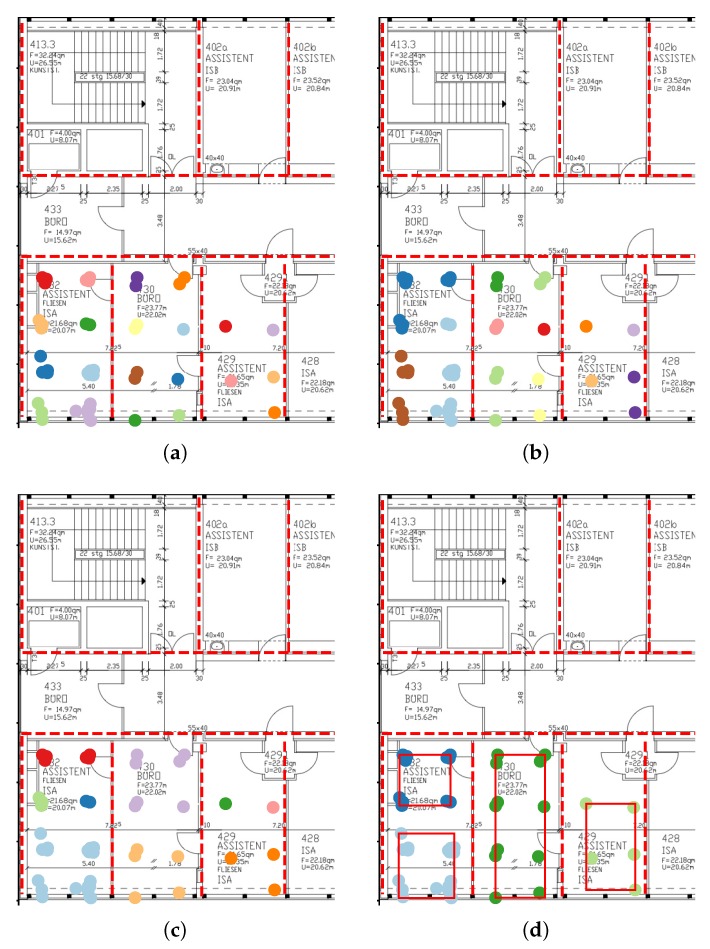
Illustration of LDCE segmentation. Clusters expand over time (**a**–**c**) until all clusters have reached a size greater than the stop_size threshold (**d**).

After we have determined the clustering, we have to construct the two-dimensional shapes that represent the floor plan segmentation. Those are obtained by using the position labels of the respective cluster members. We can construct the shape by taking the bounding box around the labels, or computing the convex or concave hull. Figure 3 shows stages of an example run of LDCE. The clusters merge over time (a–c) until all clusters have at least stop_size members (d). In the example, the final segments are obtained from the bounding boxes around the labels of the class members. The pseude code of the algorithm can be found in Algorithm 1.

**Algorithm 1** LDCE floor plan segmentation1: **Inputs:**   Fingerprints: $FP=\{fp_{n}=\left(x_{n},p_{n}\right)\}$▹n=1,…,N  Walls: $W=\{\left(x_{s_{w}},y_{s_{w}},x_{e_{w}},y_{e_{w}}\right)\}$▹w=1,…,W  Main parameters: stop_size,max_eps   Distance penalties: θ,η,ζ   DBSCAN parameters: eps,minPts   Postprocessing: minMembers   2: **Initialize:**   $dist\left[fp_{u},fp_{v}\right]\leftarrow \left|\right|p_{u}-p_{v}{\left|\right|}_{2}+\theta \cdot |W_{p_{u},p_{v}}\left|+\zeta \cdot |\right|x_{u}-x_{v}{\left|\right|}_{2}$▹1≤u,v≤N  $C_{final}\leftarrow \left\{\right\}$   $C_{exp}\leftarrow DBSCAN\left(dist,eps,minPts\right)$    ▹ Main routine 3: **while**
$|C_{exp}|>1$
**and**
min_dist<map_eps
**do** 4:  $C\_dist\left[C_{i},C_{j}\right]\leftarrow \left|\right|{\bar{p}}_{i}-{\bar{p}}_{j}{\left|\right|}_{2}+\theta \cdot |W_{{\bar{p}}_{i},{\bar{p}}_{j}}\left|+\zeta \cdot |\right|{\bar{x}}_{i}-{\bar{x}}_{j}{\left|\right|}_{2}+\eta \cdot \left|C_{i,j}\right|$▹$1\leq i,j\leq |C_{exp}|$5:  min_dist←min(C_dist) 6:  $C_{m},C_{n}\leftarrow argmin\left(C\_dist\right)$ 7:  $C_{merged}\leftarrow C_{m}\cup C_{n}$ 8:  $C_{exp}\leftarrow C_{exp}\backslash C_{x},C_{y}$ 9:  **if**
$|C_{merged}|>stop\_size$
**then** 10:    $C_{final}\leftarrow C_{final}\cup C_{merged}$ 11:  **else** 12:    $C_{exp}\leftarrow C_{exp}\cup C_{merged}$ 13:  **end if** 14: **end while**   ▹ Postprocessing 15: **for all** C in $C_{exp}$
**do** 16:  **if**
|C|>minMembers
**then** 17:    $C_{final}\leftarrow C_{final}\cup C_{merged}$ 18:    $C_{exp}\leftarrow C_{exp}\backslash C$ 19:  **end if** 20: **end for**
 21: **for all** C in $C_{exp}$
**do** 22:  Add C to closest $C_{f}\in C_{final}$
**if** closer than 2·max_eps 23: **end for**   ▹ Determine final shapes 24: $P_{k}=\left\{p_{i}|\left(p_{i},x_{i}\right)\in C_{final_{k}}\right\}$▹$k=1,\ldots ,|C_{final}|$25: $A_{k}=convex\_hull\left(P_{k}\right)$ 26: **return**
*A*   

## 5. Machine Learning Model Building

The complete pipeline of ML model building comprises (1) preprocessing of the data, (2) model training and (3) model selection and evaluation. Each step is explained in the following.

### 5.1. Preprocessing

#### 5.1.1. Feature Preprocessing

The applied machine learning models require inputs of fixed dimensions. Each access point that is observed during data collection represents one dimension of the input vector. Having observed a total amount of *M* access points, we can construct a feature vector $x_{n}={\left(x_{1},\ldots ,x_{M}\right)}^{T}$, where $x_{i}$ for i=1,…,M and n=1,…,N represents the RSS value of the i-th access point of the n-th measurement. Given a collected training sample, there is not a RSS value for each access point. This can have two reasons: (1) the access point cannot be observed at the measuring position because it is out of range, or (2) the access point is in general observable for the given location, however, its RSS value could not be recorded in that specific sample. The second reason is caused by the response rate of an access point, which is correlated with the average observable RSS value for a location [45]. For both causes of unobservable access points, an artificial value has to be chosen as entry for the feature vector. A common practice, which neglects the response rate of access points, is to simply set all missing values to a low RSS value, such as -110dB. This approach is adopted in our experiments.

For gradient-based learning algorithms such as DNNs or distance-based algorithms such as k-nearest neighbor (k-NN), it is crucial to normalize or standardize each feature column [46]. This speeds up the learning phase and prevents features with a longer range to outweigh other features. It can be distinguished between feature scaling/normalization and feature standardization (z-score normalization). Scaling linearly transforms the data into the interval [0,1], while standardization transforms the data to have zero mean and standard deviation equal to one. Standardization is especially useful if the range of the features are unknown or the feature contains many outliers. For choosing the right normalization technique, we have to investigate the influence of the given map coverage. Let $AP_{a}$ and $AP_{b}$ be two access points that are far away, such that there is no location where both can be observed simultaneously. Let $area_{a}$ and $area_{b}$ be the areas where either signals of $AP_{a}$ or $AP_{b}$ are received. A map coverage that contains much more samples of $area_{a}$ does only have few samples with signal of $AP_{b}$. When standardizing the data of the map coverage, we encode a strong bias into the preprocessed data, since the feature column of $AP_{b}$ is strongly influenced by the vast amount of zero entries. Such a bias might be tolerable if the distribution of training data matches the test data distribution. However, during online localization, users might request their position mostly within $area_{b}$, which would result in worse performance. In order to prevent this bias towards the given map coverage, we simply apply column-wise feature scaling. For each AP it is likely that a sample exist which could not register any signal strength for the AP. As a conclusion, the minimum RSS value for all columns is equal to the supplementary value for missing data.

#### 5.1.2. Floor Plan Segmentation (Parameter Choice)

To obtain the class labeled set $FP_{SEG}$, we partition the floor plan with the introduced LDCE algorithm. The choice of certain parameters of the LDCE algorithm depends on the given floor plan and the spatial distribution of available training data. The parameters eps and minPts determine the starting clusters that result from the initial DBSCAN execution. They should be chosen empirically, such that the sizes of starting clusters do not exceed the *stop_size* member threshold and not too many observations are considered as noise. The value of max_eps and the wall penalty should also be chosen empirically based on the given floor plan dimensions and the amount of walls that should be allowed within segments. The penalty term η is set to 2, since higher values might yield overlapping clusters during the initial DBSCAN execution. ζ is set to the highest penalty value of 20 to avoid intersecting final clusters. After those parameters are fixed, we can vary the stop_size and minMembers parameters to obtain multiple segmentations with various granularities. An overview of the parameters can be found in Table 1. Those parameters that depend on the given test site are revisited in the corresponding Section 6.2 and Section 6.3.

#### 5.1.3. Label Preprocessing

For training of regression models, the labels consist of the set of positions $\left\{p_{n}\right\}$, n=1,…,N, where each label is a two-dimensional vector representing the position tag. In case of area classification, the labels $\left\{y_{n}\right\}$ with $y_{n}={\left(y_{1},\ldots ,y_{K}\right)}^{T}$, n=1,…,N, for the set $FP_{SEG}$ are the one-hot encoded areas of the floor plan segmentation, where $y_{i}=1\Leftrightarrow i=c_{n}$ and 0 at all other positions. K represents the amount of segments of the given floor plan segmentation $FP_{SEG}$.

### 5.2. Model Training

In the upcoming case study in Section 6, we focus on three types of supervised machine learning models that are suitable to predict the area of unknown fingerprints. After hyperparameter tuning we end up with a DNN model that has 3 hidden layers (HL) and 512 hidden units (HU) per layer and utilizes rectified linear unit (ReLU) as activation function between layers. In order to learn the conditional probability distribution p(y|x), we apply softmax activation function for the output layer together with multiclass cross-entropy loss. This choice can be derived by following a maximum likelihood approach [47]. The Adam optimizer, a variant of stochastic gradient descent (SGD), is utilized for iterative learning of the weights. To prevent overfitting, we apply early stopping, which stops the training phase if the performance on a separate validation data set does not increase for a specified amount of epochs. Furthermore, weight regularization within the loss function and dropout are applied. The complete parameterization of the tuned DNN is given in Table 2. In addition, we train a CNN with similar hyperparameters as suggested by [24], which consists of two convolutional layers of size (16 × 16), a Maxpool layer of size (8 × 8), a convolutional layer of size (8 × 8) and a Maxpool layer of size (8 × 8). In-between layers, we add dropout layers with dropping probability of 0.25 and utilize ReLu as activation function. Finally, a fully connected dense layer of size 128 is used with output softmax activation function. We found that rearranging the RSS vector as matrix with zero padding outperforms the proposed preprocessing method of [27] that utilize the PCC to reduce the dimensionality and scale the data per access point. Furthermore, we fit a SVM with RBF kernel, which we utilize as discriminative model to directly predict y.

Additionally, we select two regression models (k-NN and DNN(reg)). The DNN regression model has the same configuration as the DNN classifier but uses a linear output activation function and mean squared error as loss function. The k-NN models apply the weighted version of the algorithm and are evaluated for three values of k, namely, 2,3 and 5. To validate whether explicitly training a classifier provides valuable results, we label the regression outputs with the closest area of the floor plan segmentation during postprocessing and compare them to the output of the area classifiers.

### 5.3. Model Evaluation

For model evaluation, we require a *splitting strategy* into training and test data as well as a *metric* that indicates how well a model performs. Those are introduced for the different model types in the following. 

*Splitting strategy:*

- Area classifiers: The training data is labeled according to the computed floor plan segmentations. We apply k-fold cross validation with k=5, such that we arrive at 20% test data per fold. We utilize the stratified version to obtain a good representative of the whole data set in each split.
- Regression models: We choose a subset of testing positions by applying DBSCAN on the position labels only. Based on the resulting clusters we apply 5-fold cross validation, such that 20% of the clusters are used as testing data in each fold.

*Metric:*

As metrics, we compute error vectors for the vectors of predictions and ground truth labels. Those error vectors can be visualized via an empirical cumulative distribution function, which we will refer to as CDF in the following.

- Area classifiers: The error vector consists of the pairwise distances between the centers of the predicted areas and the ground truth areas, which is zero in case of a correct prediction. The y-intercept of the CDF corresponds to the machine learning accuracy metric (ACC). The curve yields additional knowledge about the significance of misclassification. Furthermore, we report the $F_{1}$ score (F1).
- Regression models: In case of exact position estimation, the error vector consists of the pairwise distances between predictions and ground truth positions.
- Selection via ACS: During model selection, we utilize the ACS as metric. This requires computing the class-wise $F_{1}$ scores of the predicted and ground truth areas.

## 6. Experimental Evaluation

The subsequent experimental case study targets two separate questions:

1. Does adaptive area localization based on a data-aware floor plan segmentation provide more robust results than the standard regression approach for exact position estimation? In particular, is it suited for arbitrarily collected training data via crowdsourcing?
2. When crowdsourced training data is generated continuously, the area classifier has to adapt to the current data basis. This is accomplished by recomputing the underlying floor plan segmentation and retraining a classification model on the data labeled with the corresponding areas. In this setting, is the proposed ACS suited for automatic model selection among a pool of models that provide varying performances and expressivenesses?

### 6.1. Study Design

In order to answer these questions, we conduct two experiments.

- *Static performance analysis* (Section 6.2.1 and Section 6.3.1): we compute two floor plan segmentations with varying granularities for a snapshot of collected training data. For each segmentation we train and evaluate various classification models. In addition, the performance of the proposed area classifiers is compared to standard regression models that aim at pinpointing the exact location.
- *Model selection via ACS for continuous data collection* (Section 6.2.2 and Section 6.3.2): we subdivide all available training data into 5 epochs that contain roughly the same amount of additional data to simulate the continuous data collection. For each epoch we compute a pool of floor plan segmentations, where we choose the parameters stop_size and minMembers empirically to obtain segmentations with various granularities. Subsequently, we optimize a classifier on the data labeled with the areas. The parameter λ has to be chosen according to the use case requirements. We exemplarily choose the outer bounds (0 and 1), where 0 provides high performance and low expressiveness and 1 targets models with higher expressiveness. Furthermore, λ=0.5 is chosen to select a balanced model. We demonstrate how to utilize the ACS to automatically select the optimal model for the given use case requirements.

Both experiments are conducted on two different data sets. The first one has been collected in our university building. The second one utilizes the publicly available benchmark dataset for indoor localization using crowdsourced data [48], which was captured in Tampere, Finland. In the following we report the results grouped by the different test sites.

### 6.2. Case Study: RWTH Aachen University Building

The test environment for the data that we collected by ourselves is the 4th floor of the civil engineering building of the RWTH Aachen university, Germany. The floor contains several offices and a long hall. The total area is roughly 1500 m${}^{2}$. Two smartphones (Oneplus and LG) are used to collect labeled fingerprints with continuous position tags. In a period of 9 months (from December 2018 to August 2019), a total amount of above 1000 fingerprints have been collected. The initial performance analysis utilizes the entire training data as static data set.

#### 6.2.1. Static Performance Analysis

By applying the LDCE algorithm with two different parameterizations, we obtain two floor plan segmentations, which differ in granularity.

**Figure 4:**
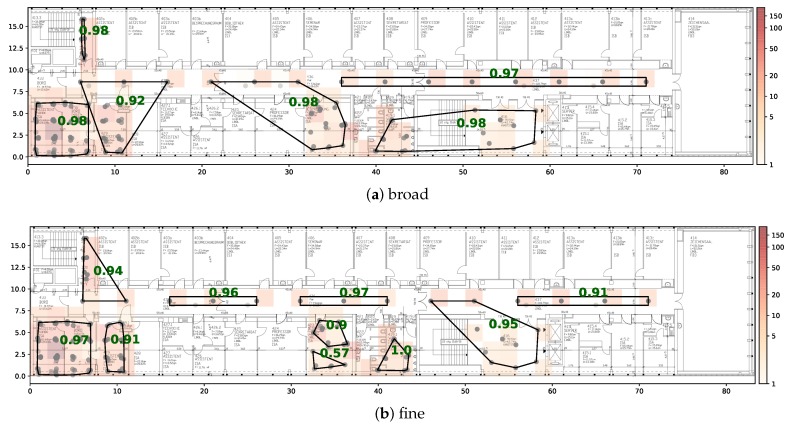
Floor plan segmentations of RWTH Aachen university building. The black lined shapes represent areas of classifier. The green numbers represent the class-wise $F_{1}$-score of the best model. The grey dots are the fingerprint locations. The amount of training data per 2 × 2 m grid cell is illustrated via the heatmap color.

The segmentations are shown in Figure 4, where the segments are represented by the shapes with black boundaries. The grey points represent fingerprint locations. We sum the amount of data per 2 × 2 m square and plot a heatmap to visualize the training data distribution. The initial DBSCAN is performed with eps=2 and minPts=3, which yields reasonably sized start clusters. We choose a wall penalty of 10 such that given max_eps=30, there will be at most 2 separating walls between merging clusters. The first segmentation (Figure 4a) sets *stop_size* equal to 80, such that clusters are excluded from the expansion set when they reach more than 80 members. The second segmentation (Figure 4b) is obtained by setting *stop_size* to 50.

We label the data set according to both segmentations and train the models described in Section 5.2 to predict the right area.

**Figure 5:**
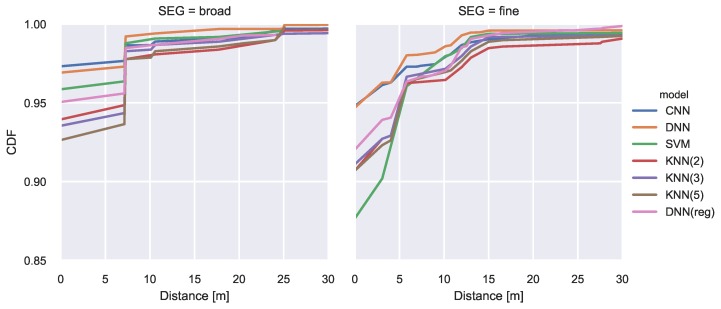
CDF of classification error. Error vector build from distances between centroids of true and predicted areas.

The resulting CDF is illustrated in Figure 5.

The CNN and the DNN achieve the best classification performance with an accuracy of above 97% on the broad segmentation and almost 95% on the finer segmentation. While the SVM achieves acceptable results for the broad segmentation its performance significantly decreases when using a finer segmentation. All regression model results are mapped to the closest class. They achieve lower performance than the CNN and DNN classifiers. A comprehensive overview of the model comparison can be found in Table 3. The lowest mean error is achieved by the DNN classifier with values of 0.43m and 0.66m respectively. For illustration purposes we plotted the class-wise $F_{1}$ score of the best performing model as green numbers for each segment in Figure 4.

In addition, we evaluate the performance of training a standard regression model for exact position estimation.

**Figure 6:**
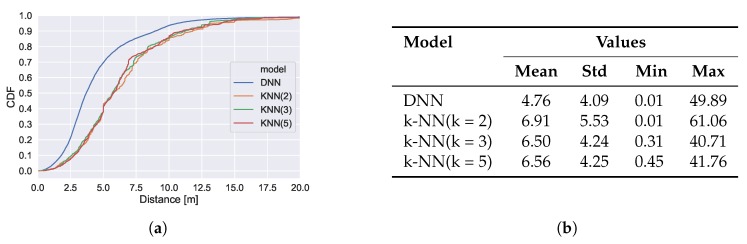
Performance of regression models. (**a**) shows the CDF of the prediction errors and (**b**) holds mean, standard deviation, minimal and maximal error.

The results are presented in Figure 6. The best regression model (DNN) guarantees that in 95% of the cases, the estimated position will not differ more than 10 m. In comparison the area classification models guarantee a correct area prediction in 95% of the cases and thus achieve more robust results. This is achieved by lowering the expressiveness and utilizing the knowledge about available training data.

#### 6.2.2. Model Selection via ACS

In the following we present the results when applying the ACS for model selection as described in Section 6.1.

**Figure 7:**
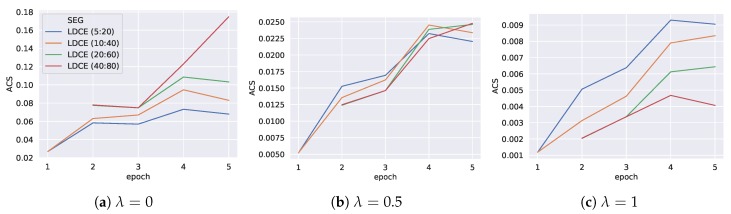
Area classification score (ACS) for three choices of λ. Per epoch the model with the highest score is chosen. The legend shows the (minMembers: stop_size) parameters used during segmentation. The other parameters of LDCE are chosen as presented in Table 1.

Figure 7 shows the ACS score of the trained models on the pool of segmentations for the three choices of λ. The figure is interpreted by fixing a choice for λ depending on the use case. At each epoch, we can now deliver the model with the highest ACS, since it provides the best balance between expressiveness and performance. Note that for the first two epochs, the segmentations obtained from stop_size={60,80} result in a single cluster, since too few data is available and are thus discarded. When inspecting the score for λ=0.5, we see that at the second and third epoch, we would use the segmentation obtained by *LDCE (5:20)*, while in epoch four the highest score is achieved on *LDCE (10:40)*. Finally, for the last epoch, the classifier that was optimized on *LDCE (40:80)* is selected.

The changes in ACS are discussed epoch-wise in the following. While epoch 1 contains only training data of the lower left offices, in epoch 2 additional training data along the hall has been collected. This allows for additional areas. *LDCE (5:20)* yields much more new segments among the hall, which causes the high increase for λ=1. For λ=0, those small segments do not affect the score, however, the achieved class-wise $F_{1}$ score does, which is slightly lower for *LDCE (10:40)*. Between epoch 2 and 3, only few new areas are covered, however, the lower left offices feature additional data. *LDCE (20:60)* and *LDCE (40:80)* are equal, which can also be observed from their similar ACS values. In *LDCE (10:40)*, the lower offices have already been split in epoch 2, which yielded a bad performance. The additional data allows for improved model performance, which explains the increased ACS. Between epoch 3 and 4, only data in previously uncovered areas is added. This causes an increased ACS value for all segmentations and λ values. For the broadest segmentation *LDCE (40:80)*, the previous areas remain the same, while the other segmentations adopt a finer granularity. Therefore, the highest relative increase for λ=0 is observed for *LDCE (40:80)*. Between epoch 4 and 5, no additional areas are covered with training data. However, segmentation *LDCE (40:80)* rearranges its area shapes, such that the total covered area increases. While the class-wise $F_{1}$ scores remain roughly the same, this causes the jump in ACS value for λ=0. The other segmentations remain mainly unchanged, since only the $F_{1}$ scores of the models slightly change.

### 6.3. Case Study: Tampere, Finland

In addition to the data collected by ourselves, we evaluate our approach on a publicly available fingerprinting dataset that was generated via crowdsourcing [48]. The original dataset consists of 4648 fingerprints collected by 21 devices in a university building in Tampere, Finland. The fingerprints are distributed over five floors, while the 1st floor contains the highest sample density. Therefore, we select the data of the 1st floor as subset to conduct our experiments.

#### 6.3.1. Static Performance Analysis

**Figure 8:**
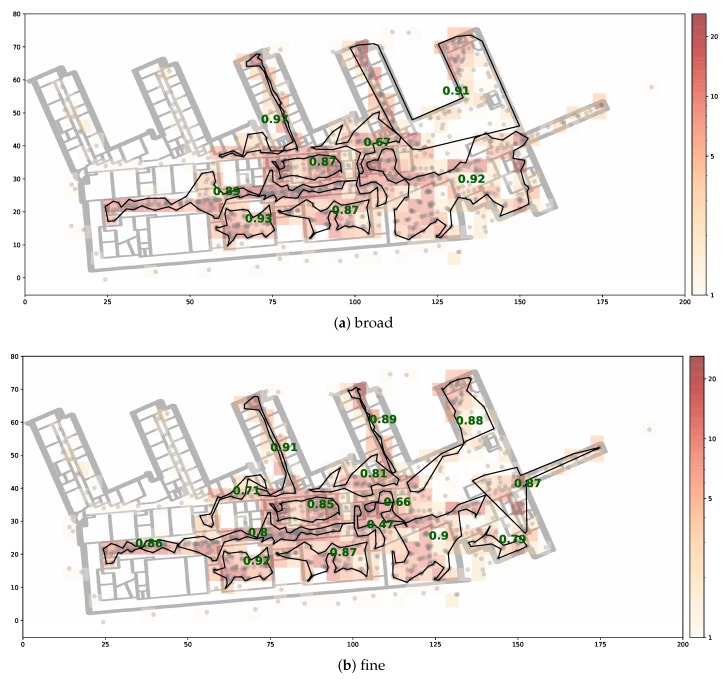
Floor plan segmentations of 1st floor of public dataset [48]. The black lined shapes represent areas of the classifier. The green numbers represent the class-wise $F_{1}$-score of the best model. The grey dots are the fingerprint locations. The amount of training data per 4x4m grid cell is illustrated via the heatmap color.

Using the entire data collected on the 1st floor, we construct two floor plan segmentations based on the LDCE algorithm, which can be found in Figure 8. The initial DBSCAN is performed with eps=5 and minPts=3. Note that in contrast to the other site, we slightly increase the eps parameter to obtain reasonably sized start clusters. This is justified because the overall training data distribution is more sparse and the map has more than 5 times the extent of the other test site. Following the same logic, we increase the max_eps parameter to 50. We use the same penalties as before but lowered the wall penalty to 5, since we want to allow clusters to span several office rooms. The remaining parameters can be found in Table 1. The broad segmentation was obtained by choosing a *stop_size* of 100 and for the fine segmentation we set *stop_size* equal to 60.

**Figure 9:**
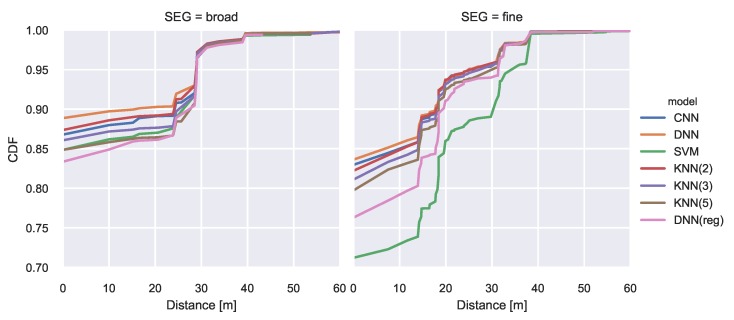
CDF of classification error. Error vector build from distances between centroids of true and predicted areas.

The dataset is published with a predetermined train test split, which consists of 20% training data and 80% testing data. When plotting the training data of the 1st floor, we noted that only a single region contains training samples, which makes the proposed split impractical. Therefore, we apply the splitting strategy described in Section 5.3. The CDF of the class-wise error vectors is presented in Figure 9. Similar to the other dataset, the DNN classification models achieve the best results independent of the segmentation. On the broad segmentation, an accuracy of 89% is reached and in 97% of the cases the predicted centroid of the area is less than 30 m off from the centroid of the true area. A comprehensive overview of the individual model performance can be found in Table 4. The DNN achieves the lowest mean centroid error and has the lowest standard deviation. The prediction performance with respect to individual areas is illustrated in Figure 8. The green numbers represent the class-wise $F_{1}$ scores that the best model achieved.

**Figure 10:**
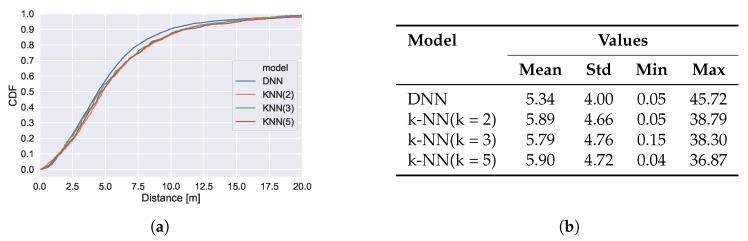
Performance of regression models. (**a**) shows the CDF of the prediction errors and (**b**) holds mean, standard deviation, minimal and maximal error.

For comparison with exact position estimation, we evaluate the performance of training standard regression models. The results are presented in Figure 10. While the DNN regression model achieves an error below 10 m with 90% probability, we achieve a correct area prediction in ~90% of the cases on the broad floor plan segmentation. Thus, for the goal of coarse localization the area classifiers provide higher guarantees.

#### 6.3.2. Model Selection via ACS

**Figure 11:**
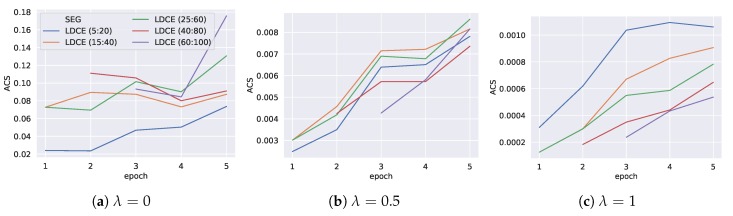
Area classification score (ACS) for three choices of λ. Per epoch the model with the highest score is chosen. The legend shows the (minMembers: stop_size) parameters used during segmentation. The other parameters of LDCE are chosen as presented in Table 1.

In the following we present the results when applying the ACS for model selection as described in Section 6.1. Figure 11 illustrates the obtained ACS scores of the trained models on the pool of segmentations for the three choices of λ. Using the ACS as selective feature, we can state the following observations. For λ=0 (high performance), the model trained on *LDCE (15:40)* is chosen for the first epoch and *LDCE (40:80)* is selected for the second and third epoch. For the entire training data the classifier trained on *LDCE (60:100)* is chosen. For λ=0.5 (balance between expressiveness and performance), *LDCE (15:40)* provides the selected segmentation for the first four epochs and is replaced by the slightly broader segmentation *LDCE (25:60)* in the last epoch. The highest expressiveness is given for λ=1, which selects the model trained on the finest segmentation *LDCE (5:20)* for all epochs.

In the following the ACS graphs are analyzed epoch-wise. In the first epoch *LDCE (25:60)* and *LDCE (15:40)* consist of only two broad segments, on which the models achieve the same class-wise $F_{1}$ scores. This can be observed, since both have the same scores for a fixed λ value. Since they cover a larger total area than the finer *LDCE (5:20)*, they score higher for low and medium λ values. However, the larger number of segments of *LDCE (5:20)* causes the higher ACS value for λ=1. In epoch 2 *LDCE (5:20)* adds the most additional segments, while the number of added segments is the same for *LDCE (15:40)* and *LDCE (25:60)*. This explains the scores observed for λ=1. While for the three segmentations the number of segments increases, high class-wise $F_{1}$ scores can be maintained for *LDCE (15:40)* and *LDCE (25:60)*. However, the finest segmentation *LDCE (5:20)* sacrifices performance for expressiveness and thus scores lower for λ=0.5. For λ=0 the score does not change much, since the total covered area remains mostly constant. However, *LDCE (40:80)*, which is present in epoch 2 for first time, covers a much wider total area, since it only consists of few large segments and therefore scores considerably higher for λ=0. Between epoch 2 and 3, data is collected in previously uncovered areas, which allows for finer segmentations independent of the chosen parameters. This can be observed by the significant increase in ACS for λ={0.5,1}. On the contrary, between epoch 3 and 4, mostly data within previously covered areas is collected, which allows for slightly higher performance. Finally, in the last epoch, the segmentations change again, while especially *LDCE (60:100)* computes a segmentation that covers a much larger total extent than the other segmentations. This explains the high increase in ACS value for λ=0.

## 7. Discussion

In the following the findings of our work are discussed. The results of the case study are analyzed with emphasis on the proposed concepts. Subsequently, the benefits of adaptive area localization are highlighted in comparison to existing solutions. And finally, potential applications of the proposed concept are described.

### 7.1. Case Study Results

*Model performance:*

Independent of the test site, the DNN area classifiers outperformed all other models with respect to standard classification metrics, such as accuracy and $F_{1}$ score. The $F_{1}$ metric indicates that the model provides high precision and recall scores, which means that each individual area is detected properly and in case it is selected the prediction is trustable. CNN models are especially useful to learn tasks where inputs are locally connected, such as adjacent pixels in images [49]. When randomly arranging the access point vector as a matrix, it cannot be claimed that a comparable relation between adjacent matrix entries exists. Therefore, the additional feature extraction should not provide any benefits, which is empirically demonstrated by the results. The SVM model can only be used as multi-class classifier by training several individual classifiers and following a certain voting scheme. We applied the one-vs-one strategy, which results in K(K−1)/2 classifiers if we want to detect K areas. Besides, the high computational effort, the results are worse than a simple k-NN classifier, which is also observed in [50]. 

*LDCE floor plan segmentation algorithm:*

During the second experiment, it was demonstrated that the proposed LDCE algorithm is capable of providing a pool of segmentations with various granularities. Those can be utilized in combination with the proposed ACS to select the best area classifier with respect to the right balance between expressiveness and performance. The algorithm requires certain parameters to be chosen empirically based on the given site. 

*ACS model selection metric:*

The effect of λ on the ACS was theoretically evaluated and demonstrated for three values in the experiments. However, explicit values cannot be associated with qualitative terms, yet. In particular, it cannot be stated which exact value is optimal for a certain use case. However, the ACS is lazily computed. Once an area classifier has been trained, its ACS can be computed for several choices of λ by utilizing the stored prediction and ground truth vectors. This means that it is computationally inexpensive to compute the ACS for a pool of trained models and a large set of λ values. An initial λ value is guessed. When the retrieved model does not meet the requirements, λ can be adjusted to match the right balance between expressiveness and performance.

### 7.2. Adaptive Area Localization

Area localization has been proposed for large-scale deployments of fingerprinting-based solutions or when the data quality does not allow for exact position estimation. The objective is to provide higher positioning guarantees by lowering the expressiveness of the model. In related work, the segmentation during area classification features two characteristics [17,18,19,20]:

- It is determined independent of the available training data.
- It is statically determined, mostly prior to data collection.

Both features are unfavorable when working with crowdsourced data that is continuously collected and solutions to apply area localization in such settings are currently missing in the literature. Crowdsourced data collection results in a spatially non-uniform data distribution [15]. Training a classifier on data where certain areas (classes) feature only few or no samples results in poor performance. A segmentation that is determined independent of the training data might result in such sparsely covered areas. Therefore, we introduce the concept of data-aware floor plan segmentation and propose the LDCE algorithm that computes such a segmentation. A data-aware floor plan segmentation introduces a trade-off between expressiveness and performance, which has not been quantified in the literature, yet. However, such a quantification is required to measure how well an area classifier performs given that the underlying segmentation is not static. Therefore, we propose the ACS that captures this trade-off. Furthermore, during crowdsourcing, data is accumulated over time. The segmentation determined for a given snapshot of data might become unfavorable once additional data has been collected. It is crucial to regularly recompute the segmentation into areas. In summary, our proposed concepts enable area localization for crowdsourced data and we empirically demonstrate that this achieves higher reliability than exact position estimation. The model adapts to the accumulating training data and finds the right balance between expressiveness and performance.

### 7.3. Potential Applications

Depending on the use case, localization systems might have distinct requirements. A system with the objective to provide proximity based services (e.g., inside a shopping mall [17]) requires a coarse-grained position estimation with high guarantees. In contrast, a localization system utilized for navigation of people with visual impairments might benefit from a more fine-grained position estimation. Given a base of crowdsourced training data, our approach allows to automatically construct area localization models for any required tradeoff between expressiveness and performance. Furthermore, it adapts to the accumulating training data that results from continuous crowdsourced data collection. To the best of our knowledge, generating such adaptive localization models based on fingerprinting has not been proposed in the literature, yet.

In addition, absolute location information can be merged with systems that iteratively determine the position of a user such as PDR. WLAN fingerprinting is already employed in sensor fusion solutions [51,52,53]. The granularity and level of guarantee of the fingerprinting model might impact initialization and convergence time of the fused model. With our approach, the fingerprinting-based localization model with the optimal granularity in that regards can be trained and deployed in the fused model.

## 8. Conclusions

In this work, we propose the concept of adaptive area localization to achieve reliable position estimations using crowdsourced data that is accumulated over time. Existing area localization solutions employ a static segmentation into areas that is independent of the available training data. This approach is not applicable for crowdsoucred data collection, since it features an unbalanced spatial training data distribution that changes over time. To solve this, we propose the LDCE algorithm that computes data-aware floor plan segmentations with various granularities. The underlying segmentation influences the model performance as well as its expressiveness. We introduce the ACS to select the area classifier that provides the best trade-off between them. With those concepts, we can now regularly compute a pool of segmentations and train classifiers on the data labeled with the corresponding areas. We select the best model with the ACS and deploy it for localization.

The proposed concepts are validated on a self-collected as well as on a publicly available crowdsourced data set. We demonstrate that the proposed area classifiers provide higher positioning guarantees than models for exact position estimation. Furthermore, we show that they adapt to the accumulating data base. In future work, we want to utilize PDR techniques and sensor fusion to automate the data collection process and to enhance the positioning quality during localization. In addition, our approach is not limited to WLAN RSS fingerprinting, but can be extended to support magnetic and light sensors [54,55] or bluetooth [56], which we want to demonstrate in future work.

## Figures and Tables

**Table 1:** Parameter choice of LDCE for experiments.

Data Set	Main		Postprocessing	Penalties			DBSCAN	
	stop_size	max_eps	minMembers	θ	ζ	η	eps	minPts
RWTH Aachen	{80, 50}	30	{40,20}	10	2	20	2	3
Tampere, Finnland	{100, 60}	50	{60, 40}	5	2	20	5	3

**Table 2:** DNN model hyperparameter configuration.

HU	HL	Dropout	Reg. Penalty	lr	Batch	Epochs	Loss	Activation	Optimizer
512	3	0.2	0.06	0.0007	32	200	Cat. cross-entropy	ReLU	Adam

**Table 3:** Performance of classification models on both segmentations. The upper three models are explicitly trained to predict one of the underlying areas, while the other models (reg->class) are regression models where we assign the closest area of the regression prediction during postprocessing.

Segmentation	Model	Parameter	Area Center Error				Classification	
			Mean	Std	Min	Max	ACC	F1
broad	CNN		0.43	3.28	0.0	47.42	0.97	0.97
	DNN		0.32	2.17	0.0	47.42	0.97	0.97
	SVM		0.54	3.46	0.0	45.25	0.96	0.95
	k-NN (reg- > class)	k = 2	0.85	4.36	0.0	49.95	0.94	0.93
	k-NN (reg- > class)	k = 3	0.82	4.30	0.0	49.95	0.94	0.93
	k-NN (reg- > class)	k = 5	0.87	3.94	0.0	49.95	0.93	0.92
	DNN (reg- > class)		0.56	2.88	0.0	25.09	0.95	0.95
fine	CNN		0.66	4.18	0.0	55.12	0.95	0.91
	DNN		0.54	3.74	0.0	59.90	0.95	0.91
	SVM		1.12	4.80	0.0	59.90	0.88	0.79
	k-NN (reg- > class)	k = 2	1.15	5.47	0.0	59.90	0.91	0.84
	k-NN (reg- > class)	k = 3	0.99	4.94	0.0	59.90	0.91	0.87
	k-NN (reg- > class)	k = 5	1.00	4.54	0.0	48.34	0.91	0.86
	DNN (reg- > class)		0.71	3.07	0.0	42.50	0.92	0.87

**Table 4:** Performance of classification models on both segmentations. The upper three models are explicitly trained to predict one of the underlying areas, while the other models (reg->class) are regression models where we assign the closest area of the regression prediction during postprocessing.

Segmentation	Model	Parameter	Area Center Error				Classification	
			Mean	Std	Min	Max	ACC	F1
broad	CNN		3.70	10.15	0.0	69.26	0.87	0.86
	DNN		3.21	9.60	0.0	69.26	0.89	0.88
	SVM		4.30	10.92	0.0	65.84	0.85	0.83
	k-NN (reg- > class)	k = 2	3.55	10.02	0.0	69.26	0.87	0.86
	k-NN (reg- > class)	k = 3	3.97	10.48	0.0	69.26	0.86	0.85
	k-NN (reg- > class)	k = 5	4.34	10.84	0.0	65.84	0.85	0.83
	DNN (reg- > class)		4.62	11.17	0.0	65.84	0.83	0.81
fine	CNN		3.65	9.11	0.0	90.47	0.83	0.81
	DNN		3.53	9.00	0.0	91.75	0.84	0.81
	SVM		7.00	12.36	0.0	100.44	0.71	0.56
	k-NN (reg- > class)	k = 2	3.72	9.12	0.0	90.47	0.82	0.79
	k-NN (reg- > class)	k = 3	3.95	9.30	0.0	90.47	0.81	0.77
	k-NN (reg- > class)	k = 5	4.25	9.55	0.0	69.79	0.80	0.76
	DNN(reg)		5.00	10.07	0.0	69.79	0.76	0.72
